# Rare germline mutation and MSH2-&MSH6 + expression in a double primary carcinoma of colorectal carcinoma and endometrial carcinoma: a case report

**DOI:** 10.1186/s13000-024-01447-8

**Published:** 2024-01-31

**Authors:** Tiansong Zhang, Xiaoqiang Huang, Wenjie Liu, Xiulan Ling, Zhenping Su, Mengwei Huang, Shuanlong Che

**Affiliations:** 1 Department of Obstetrics and Gynecology, Guangzhou Women and Children’s Medical Center, Guangzhou Medical University, Guangzhou Guangdong, 510623 China; 2 Meizhou Maternal and Child Health Care Hospital, Meizhou, 514000 Guangdong China; 3 Guangzhou KingMed Center for Clinical Laboratory Co. Ltd, Guangzhou, China; 4 Shenzhen KingMed Medical Laboratory, Shenzhen, China

**Keywords:** Double primary carcinoma, Deficient mismatch repair gene, Novel mutation, Lynch-Like syndrome

## Abstract

**Background:**

Multiple primary malignancies are rare in cancer patients, and risk factors may include genetics, viral infection, smoking, radiation, and other environmental factors. Lynch syndrome (LS) is the most prevalent form of hereditary predisposition to double primary colorectal and endometrial cancer in females. LS, also known as hereditary nonpolyposis colorectal cancer (HNPCC), is a common autosomal dominant condition. Pathogenic germline variants in the DNA mismatch repair (MMR) genes, namely MLH1, MSH2, MSH6, and PMS2, and less frequently, deletions in the 3’ end of EPCAM cause LS. It manifested itself as loss of MMR nuclear tumor staining (MMR protein deficient, dMMR).

**Case presentation:**

This case study describes a double primary carcinoma in a 49-year-old female. In June 2022, the patient was diagnosed with highly to moderately differentiated endometrioid adenocarcinoma. The patient’s mother died of esophageal cancer at age 50, and the father died of undefined reasons at age 70. Immunohistochemical stainings found ER (++), PR (++), P53 (+), MSH2 (-), MSH6 (+), MLH1 (+), and PMS2 (+). MMR gene sequencing was performed on endometrial tumor and peripheral blood samples from this patient. The patient carried two pathogenic somatic mutations in the endometrial tumor, MSH6 c.3261dupC (p.Phe1088LeufsTer5) and MSH2 c.445_448dup (p.Val150fs), in addition to a rare germline mutation MSH6 c.133G > C (p.Gly45Arg). Two years ago, the patient was diagnosed with moderately differentiated adenocarcinoma in the left-half colon. Immunohistochemical stainings found MSH2(-), MSH6(+), MLH1(+), and PMS2(+) (data not shown).

**Conclusions:**

In the case of a patient with double primary EC and CRC, a careful evaluation of the IHC and the genetic data was presented. The patient carried rare compound heterozygous variants, a germline missense mutation, and a somatic frameshift mutation of MSH6, combined with a novel somatic null variant of MSH2. Our study broadened the variant spectrum of double primary cancer and provided insight into the molecular basis for abnormal MSH2 protein loss and double primary carcinoma.

**Supplementary Information:**

The online version contains supplementary material available at 10.1186/s13000-024-01447-8.

## Background

Multiple primary cancers (MPCs), also known as multiple primary malignancies, are defined as the presence of two or more different primary cancers in the same patient occurring in the same or different organs or tissues [1, 2].

Lynch syndrome (LS) is the most prevalent form of hereditary predisposition to multiple primary tumors, most notably primary colorectal carcinoma and endometrial cancer in females [3].

LS, also known as Hereditary Nonpolyposis Colon Cancer (HNPCC), is an autosomal dominant inherited disease. LS patients can develop multiple extraintestinal malignant tumors, such as cancers in the ovaries, stomach, small intestine, liver, and biliary system [4]. In 1966, Henry T. Lynch of the United States first reported Lynch syndrome. The incidence rate of Lynch syndrome in the population is 1/200-1/1000 [5–7]. The lifetime risk of developing cancer in males with Lynch syndrome is up to 90%, while in females it is about 69% [8, 9]. Lynch syndrome is mainly caused by germline mutations in the mismatch repair gene (MMR). Mutations in MMR genes cause both accumulation of replication errors in microsatellite sequences and deficient expression of the proteins encoded by the mutated MMR genes in the tumor.

The current detection methods include immunohistochemical staining (IHC), PCR, and NGS. IHC mainly detects the expression levels of four proteins, MLH1, MSH2, MSH6, and PMS2. The PCR method is most commonly used in microsatellite sequences, and mainly includes two single nucleotide repeat sites (BAT-25, BAT-26, NR21, NR24, and NR27) and three dinucleotide repeat sites (D2S123, D5S346, and D17S250) [10]. Next-generation sequencing (NGS) is a powerful and versatile technology offering many possibilities and advantages compared to the gold standard PCR and capillary electrophoresis approach. NGS could analyze both MSI status and genetic alterations in MMR genes, which makes NGS more and more widely used [11]. IHC detection for MMR proteins is commonly used for prescreening for suspected Lynch syndrome patients in most genetic testing laboratories according to a widely used algorithm [12].

Patients with germline mutations in the MSH2 gene usually exhibit concurrent deletion of hMSH6. Similarly, mutations in the MLH1 gene usually also show deletion of both MLH1 and PMS2. Germline mutations in the MSH6 or PMS2 gene result in separate deletions of corresponding proteins, while hMSH2 and MLH1 retain normal expression. However, cases of MSH2 deficiency and MSH6 normal expression in immunohistochemistry are very rare. This article will report a rare case of metachronous double primary carcinoma with loss of MSH2 protein and normal MSH6 expression, and probe the molecular mechanisms behind it.

## Case presentation

**Fig. 1:**
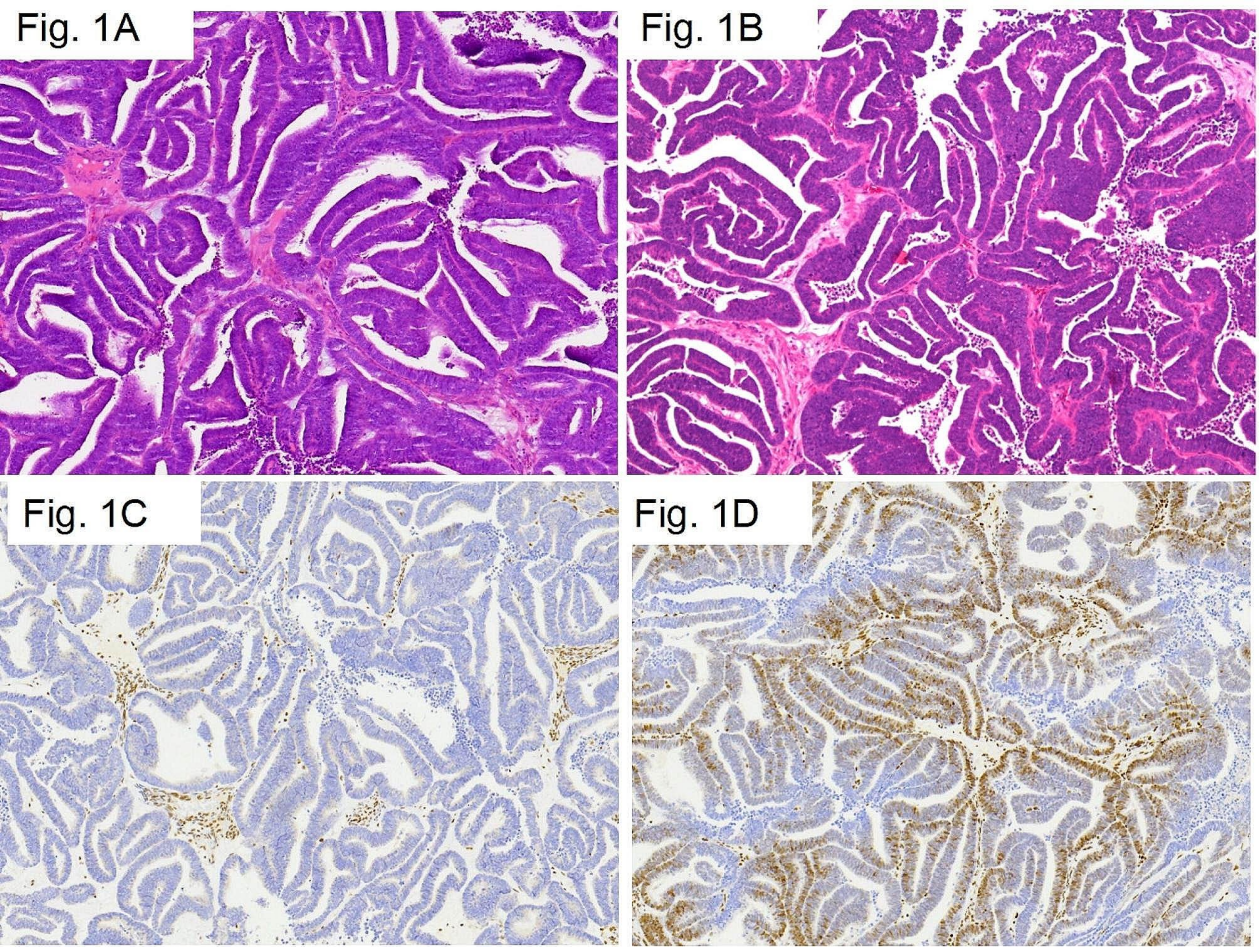
**A**, Moderate differentiation endometrioid adenocarcinoma in the uterus, with irregular glandular and papillary tumor cells excluded. There are many dead bodies in the cavity, 100x (HE). **B**, Moderate differentiation endometrioid adenocarcinoma in the uterus, with significant cell heterogeneity, 200x (HE). **C**, Moderate differentiation endometrioid adenocarcinoma in the uterus, immunohistochemical MSH2 protein expression loss, 40x (IHC). **D** Moderate differentiation endometrioid adenocarcinoma in the uterus, immunohistochemical MSH6 protein expression normal, 100x (IHC)

A 49-year-old female was diagnosed with left-half colon cancer who underwent surgical treatment in Oct 2020 in the Third Affiliated Hospital of Sun Yat-sen University (Yuedong Hospital). Pathological results showed moderately differentiated colon adenocarcinoma with cancer tissue infiltrating the superficial muscle layer of the intestinal wall, while with no definite intravascular cancerous embolus or nerve invasion (pT2N0M0). No metastatic cancer was seen in the central group of lymph nodes and intermediate group of lymph nodes. IHC staining of the MMR protein revealed lack of expression of the hMSH2 protein, whereas hMSH6 was normally expressed (data not shown). The patient was treated with surgery and no drug therapy. In June 2022, the patient was diagnosed with endometrioid adenocarcinoma. Surgery pathological showed the endometrioid adenocarcinoma differentiated in the uterus (moderately differentiated, stage IA), invasion of the superficial muscle layer (< 1/2 muscle wall) without vascular and nerve invasion. No metastatic cancer was seen in the lymph nodes. According to the 2023 version of FIGO staging, this patient is classified as IA2. And immunohistochemistry staining in the uterine corpuscles tumor revealed MSH-2 (-), MSH-6 (+) (Fig. 1E and F, respectively), ER (++), PR (++), and P53 (+) (Supplementary Fig. 1). The patient was treated with total hysterectomy and bilateral adnexectomy under laparoscopic, and no drug therapy. After morphological standard subtyping, the patient’s endometrioid tumor and peripheral blood samples were subjected to NGS using with a Lynch syndrome panel. On 2022-12-05, the patient went for a follow-up examination of CRC 2 years after surgery. CT scan for the pelvis, abdomen, and chest revealed that the anastomosis did not show any significant abnormality. On 2022-12-02, the patient went to Meizhou Maternal and Child Health Care Hospital for follow-up examination of endometrial cancer. Examinations showed normal vulvovaginal development, vaginal patency, little intravaginal discharge, and good healing of vaginal stumps. CEA, AFP, and CA125 were all negative.

### Histopathological review of tissues

A histopathological diagnosis of the tumor tissue was performed following the World Health Organization (WHO) standards. Hematoxylin and eosin-stained slides of biopsy or surgery specimens were reviewed by an experienced pathologist. Immunohistochemistry (IHC) assays were conducted to facilitate the histopathological classification. The main steps of IHC include antigen retrieval, primary antibody binding, secondary antibody binding and 3,3’ Diaminobenzidine (DAB) staining. The IHC staining for protein biomarkers was semi-quantitatively scored as “-” (negative, no, or < 5% positive cells), “+” (5-50% positive cells), and “++” (< 50% positive cells, considered as strongly positive).

### Targeted sequencing

A sequencing panel covering five genes that have been reported to be associated with Lynch syndrome (EPCAM, MLH1, MSH2, MSH6, PMS2) was designed and performed in the local medical laboratory. This assay uses a second-generation sequencing capture enrichment method and covers all coding regions and neighboring splice regions of these five genes and a certain length of upstream and downstream untranslated regions (UTRs). The assay detected substitutions, small insertions and deletions, and copy number variants in all coding exons and flanking regions in these five genes. DNA from both FFPE and peripheral whole blood samples were extracted with QIAamp DNA FFPE Kit and QIAamp DNA Blood Mini Kit (Qiagen, Dusseldorf, Germany) according to the standard operation procedures, respectively. DNA libraries were constructed via the KAPA Library Amplification Kit (KAPA Biosystems, Cape Town, South Africa). DNA sequencing was performed on a NovaSeq6000 platform using an XP kit (Illumina, San Diego, CA).

### Mutation calling and annotation

The generated raw data with bcl format were transferred to fastq format by base calling via bcl2fastq v2.17 while filtering the low-quality reads, removing adapters and primer sequence. Alignment to human genome reference via bwa v0.7.10 and generated the bam file, with the utilization of samtool v1.3.1 to generate index file of bam. Somatic mutations were called using the unmatched normal synthetic bam file with MuTect v2 while Annovar was utilized to perform the annotation of SNV and indel, which combined with several databases including PolyPhen2 and Mutation Toaster for further annotation with alteration frequencies, enzyme binding-site prediction, and protein functional alteration prediction.

### Clinicopathological characteristics of EC combined with CRC double primary cancer

Pathological results revealed moderately differentiated colon adenocarcinoma with cancer tissue infiltrating the superficial muscle layer of the intestinal wall, And the glandular cavity contains a large amount of mucus, with obvious cell atypia. Immunohistochemistry was performed to detect MMR protein expression and found MSH2(-), MSH6(+), MLH1(+), and PMS2(+) (data not shown). In the second cancer, endometrioid tumor showed significant cell heterogeneity (Fig. 1A and B). Immunohistochemistry staining in the uterine corpuscles tumor revealed MSH-2(-), MSH-6(+) (Fig. 1C and D, respectively), ER(++), PR(++), and P53(+) (Supplementary Fig. 1). ER and PR expressions indicated that the tumor was of endometrial origin, metastatic colon carcinoma was excluded. Based on the clinicopathological characteristics, this case is a metachronous double primary carcinoma.

### Molecular analyses

**Fig. 2:**
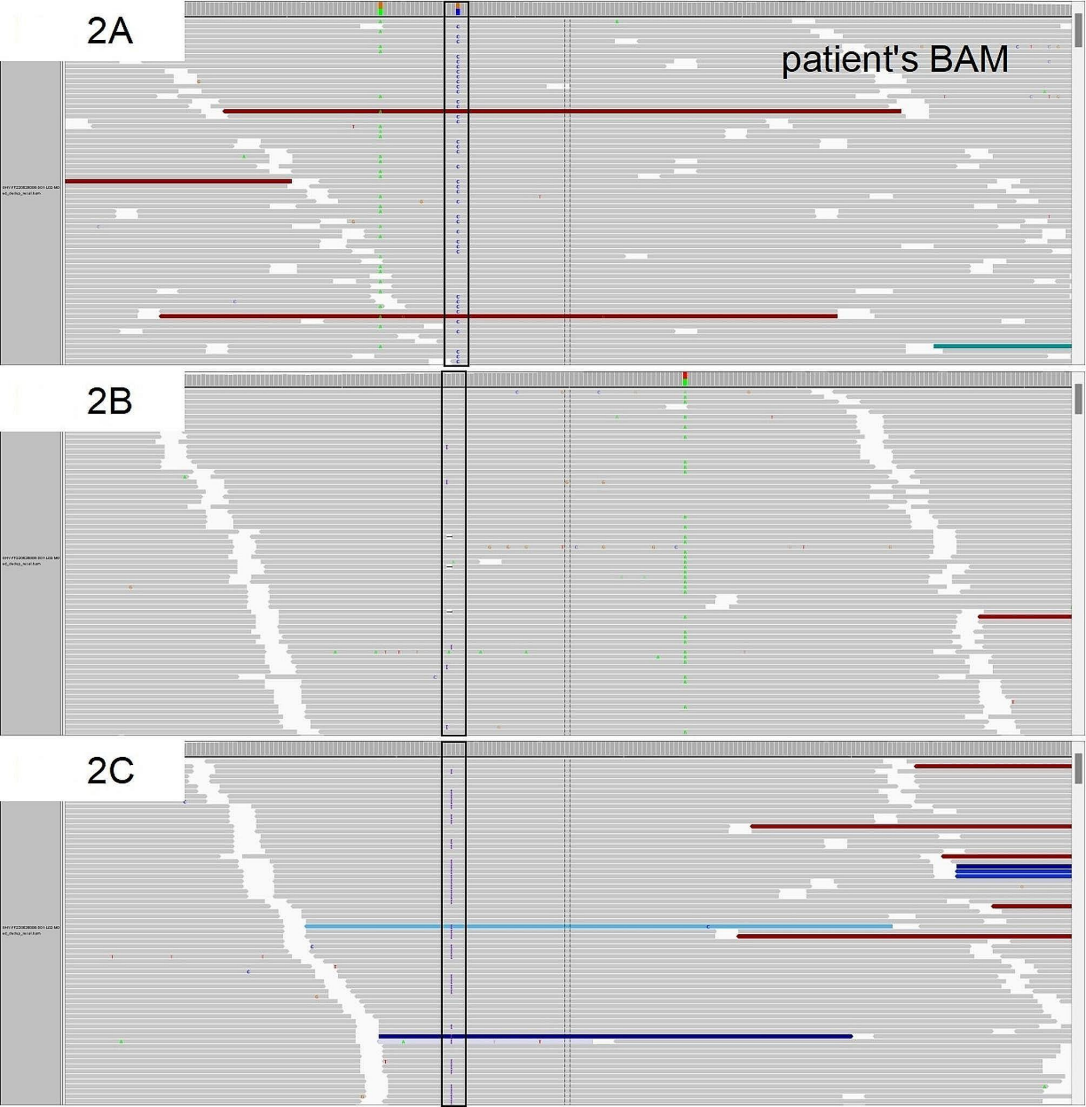
**A**, The BAM panel showed the mutation c.133G > C in MSH6 exon 1 displayed by Integrative Genomics Viewer. Black boxes indicate the mutational positions. **B**, The BAM panel showed the mutation c.3261dupC in MSH6 exon 5. **C**, The BAM panel showed the mutation c.445_448dup in MSH2 exon 3.

The proband’s mother died of esophageal cancer at 50, and her father died at 70 for undefined reasons. The patient’s peripheral blood and endometrial cancer samples underwent genetic testing.

Targeted sequencing revealed compound heterozygous variants of MMR genes in the patient endometrial specimen: germline c.133G > C (p.Gly45Arg) (allele rate, 49.9%) and somatic c.3261dupC (allele rate, 5.59%) in MSH6, and a novel somatic mutation c.445_448dup (allele rate, 42.89%) in MSH2. The MSH6 germline mutation, c.133G > C in exon 1, was also detected in the peripheral blood. The c.133G > C (p.Gly45Arg) variant was a very rare variant (only found 3 ClinVar submissions) and was predicted to be neutral, deleterious, and neutral in MutationTaster, FATHMM, and PROVEAN, respectively. ACMG classification and the evidence of pathogenicity were uncertain significance and PM2 + PP1, respectively. MSH6 c.3261dup was a rare variant and is present in the Exome Aggregation Consortium database (http://exac.broadinstitute.org) at a minor allele frequency of 0.18%. ACMG classification and the evidence of pathogenicity were pathogenic, and PVS1 + PM2 + PP5, respectively. The mutation MSH2 c.445_448dup was novel with no record in any databases. ACMG classification and the evidence of pathogenicity were likely pathogenic, and PVS1 + PM2, respectively.

## Discussion

The main issues of this case study of metachronous endometrial and colorectal carcinoma are as follows: (1) it is more prone to be Lynch-like syndrome or sporadic? (2) the molecular basis of the peculiar phenotype: loss of MSH2 protein expression but normal MSH6 protein expression.

The cause of double primary cancers is unclear and may be related to a variety of factors.

Different mechanisms have been proposed involving multiple primary cancers, such as family history, immune and genetic deficiencies, prolonged exposure to carcinogens, radiation, and chemotherapy in primary cancers, and field carcinogenesis. Studies demonstrated women with diabetes or abnormal glucose tolerance and obesity are prone to EC and CRC [13, 14]. Tianyi YE et al. found that 55.9% (19/34) of first-degree relatives of patients with double primary cancers had CRC and/or EC, suggesting that double primary cancers may be a hereditary disease [15].

Studies have shown that patients with Lynch syndrome (LS) have a significantly increased risk of developing extracolonic tumors in the endometrium, ovaries, pancreas, and prostate. In 2007, the Society of Gynecologic Oncology (SGO) [16] concluded that 20–25% of patients < 50 years of age with concurrent or heterochronous combination of EC and CRC were LS, and researchers found that 20% of patients with LS had CRC detected within 10 years after the diagnosis of EC; and 12% had EC detected within 10 years after the diagnosis of CRC [17, 18].

In the case, the patient’s deceased mother had died of esophageal cancer. The patient was physically fit and denied any history of hypertension, coronary heart disease, diabetes mellitus, viral hepatitis, tuberculosis, typhoid fever, and other infectious diseases. To identify Lynch syndrome, we performed IHC and found a loss of MSH2 expression in both EC and CRC specimens. Because the two malignancies shared a same genetic background, we preferred these were not sporadic which occur randomly. MMR gene sequencing of endometrial cancer tumors and peripheral blood in this patient was further taken.

The patient carries two pathogenic somatic mutations among the endometrial tumors, MSH6 c.3261dupC (p.Phe1088LeufsTer5) and MSH2 c.445_448dup (p.Val150fs), in addition to a rare germline mutation MSH6 c.133G > C (p.Gly45Arg). We searched the ClinVar database for the null variant MSH6 c.3261dupC and found 38 submissions. Of these, 14 were germline mutations from patients with Lynch syndrome, and one record reported a somatic mutation from a case of Lynch-like syndrome (National Center for Biotechnology Information. ClinVar; https://www.ncbi.nlm.nih.gov/clinvar/variation/VCV000089364.84). For the variant MSH2 c.445_448dup (p.Val150fs), no records were found in the ClinVar and gnomAD database. It is novel. For MSH6 c.133G > C (p.Gly45Arg), 3 submissions were found in ClinVar, all of which were germline mutations from patients with Hereditary cancer-predisposing syndrome or Lynch syndrome (National Center for Biotechnology Information. ClinVar; https://www.ncbi.nlm.nih.gov/clinvar/variation/VCV000525725.13 ).

The p.G45R variant (c.133G > C), located in coding exon 1 of the MSH6 gene, results from a G to C substitution at nucleotide position 133. The glycine at codon 45 is replaced by arginine, an amino acid with dissimilar properties. This amino acid position is not well-conserved in available vertebrate species. In addition, this mutation is predicted to be tolerated by in silico analysis. Since supporting evidence is limited at this time, the clinical significance of this alteration remains unclear.

Although loss of MSH2 protein expression was found in tumors, no germline mutations were detected in MSH2 and/or EPCAM in the patient. It does not meet the classic “second hit” theory generally that a somatic mutation in the wild-type allele of the affected MMR gene is required for triggering tumorigenesis. So, the patient does not meet the current diagnostic criteria for Lynch syndrome.

Because of the atypical family history of this patient and dMMR, we further considered lynch-like syndrome. Some patients with Lynch syndrome-associated tumors have neither high methylation of the MLH1 promoter nor germline mutations in the MMR genes but have MMR defects with clinical features similar to those of LS. This disease, which resembles LS but cannot be classified as LS, is known as lynch-like syndrome (LLS) [19]. Published data suggest that using current diagnostic methods, approximately more than half of dMMR CRC can be categorized as LLS. Similar to patients with LS, patients suspected of having LLS also had their cancer at a younger age (53.7 years s.45 years) [20].

It’s preferred that the patient has LLS, based on the following points: (1) it’s a metachronous EC and CRC, shared a common genetic background, named loss of MSH2 protein expression; (2) detected a somatic MSH2 pathogenic mutation in endometrial tumor, which was reported in a Lynch-like syndrome patient, but no germline MSH2 alterations found; (3) detected a rare germline MSH6 mutation (c.133G > C, p.Gly45Arg), which was reported in 3 patients with Hereditary cancer-predisposing syndrome or Lynch syndrome; it may be upgraded to a likely pathogenic mutation; (4) the patient had CRC at a young age of 47; (5) the patient’s mother died of cancer.

However, the possibility that the patient was sporadic cannot be excluded. To make a complete diagnosis, further investigations are needed to clarify which mutations are involved. The defective MMR protein expression in tumor tissue can be attributable to germline or somatic mutation in another DNA repair or replication-associated gene, such as BRAF, POLE/POLD1, or less frequently, MUTYH [21, 22].

Second, IHC revealed a peculiar phenotype: loss of MSH2 protein expression but normal MSH6 protein expression. In MMR protein heterodimer pairing, MSH6 and MSH2 are formed dimers, which are prerequisites for the activation of DNA repair function [23]. It is generally thought that MSH2 and MSH6, are co-expressed or co-deleted. The absence of MSH2 subsequently causes the degradation of cellular MSH6 because MSH6 has no other dimerization partners apart from MSH2. That retained MSH6 expression in the case of MSH2 loss is uncommon. This condition occurred when the tests were performed on different lesions and in different hospitals, and we excluded factors due to experimental errors. The molecular basis of dMMR from this Lynch-suspected patient is atypical but educational. A somatic variant MSH2 c.445_448dup (p.Val150fs) was found in the patient. Interestingly, it’s a novel mutation, and no biallelic somatic MSH2 inactivation was found. As any identifiable pathogenic germline mutation in MSH2 and/or EPCAM was absent, reasons for the loss of MSH2 immunohistochemical staining were unexplained. We considered other reasons except MSH2 null variants, such as the loss of ligand binding.

Hypotheses were pursued that somatic deletion of the MSH2-MSH6 region and hindered MSH2/MSH6 dimerization would lead to abnormal IHC patterns [24, 25]. In this case, one explanation would be proposed that the somatic frameshift mutation, c.3261dupC (p.Phe1088LeufsTer5), within the (C)8 tract in exon 5 of the MSH6 gene attributed to the loss of the MSH2-MSH6 interaction region and reduced MSH6 protein expression, secondary to defective binding to MSH2 protein (Supplementary Fig. 2) [26]. When paired with the null variant MSH2 p.Val150fs, this somatic frameshift mutation of MSH6-C8 provides a molecular basis for the absence of MSH2 protein expression [27, 28].

## Conclusions

In the case of a double primary EC and CRC patient, a careful evaluation of the IHC and the genetic data had been presented. The case carried rare compound heterozygous variants, a germline missense mutation, and a somatic frameshift mutation of MSH6, combined with a novel somatic null variant of MSH2. Our study broadened the variant spectrum of double primary cancer, and gained insight into the molecular basis for abnormal MSH2 protein loss and double primary carcinoma.

But there are also some limitations. Since the parents are deceased, the trios analysis was not performed. Due to a lack of funding to support the research, the patient and her siblings could not afford to take more tests. So, the relationship between the clinical features and the rare germline missense mutation of MSH6 could not be well characterized. Due to a lack of experimental evidence, the exact mechanism of the abnormal MSH2 protein loss cannot be elucidated molecularly.

### Electronic supplementary material

Below is the link to the electronic supplementary material.

Supplementary Material 1

## Data Availability

All data generated or analyzed during this study are included in this published article.

## References

[1] Jiao F, Hu H, Wang LW (2013). Quadruple primary malignancy patient with Survival Time more than 20 years. World J Gastroenterol.

[2] Coyte A, Morrison DS, McLoone P (2014). Second primary cancer risk—the impact of applying different definitions of multiple primaries: results from a retrospective population-based cancer registry study. BMC Cancer.

[3] Curtius K, Gupta S, Boland CR (2022). Review article: lynch syndrome-a mechanistic and clinical management update. Aliment Pharmacol Ther.

[4] The Committee of Colorectal Cancer, Chinese Society of Clinical Oncology;Genetics Group of the Committee of Colorectal Cancer, China Anti-cancer Association;Genetics Committee of the Committee of Colorectal Cancer, Chinese Medical Doctor Association. Consensus on detection of microsatellite instability in colorectalcancer and other related solid tumors in China [J]. J Practical Oncol, 2019(005):034: 381–389.

[5] Boland PM, Yurgelun MB, Boland CR (2018). Recent progress in Lynch syndrome and other familial colorectal cancer syndromes. CA Cancer J Clin.

[6] Lynch HT, Lynch PM, Lanspa SJ, Snyder CL, Lynch JF, Boland CR (2009). Review of the Lynch syndrome: history, molecular genetics, screening, differential diagnosis, and medicolegal ramifications. Clin Genet.

[7] Frankel W, Arends M, Frayling IM, Nagtegaal ID. Lynch Syndrome: genetic tumour syndromes of the Digestive System. World Health Organization Classification of Tumours of the Digestive System. 5 ed. IARC Press; 2019.

[8] Ladabaum U, Ford JM, Martel M, Barkun AN. American Gastroenterological Association Technical Review on the diagnosis and management of Lynch Syndrome. Volume 149. Gastroenterology; 2015. pp. 783–813. 10.1053/j.gastro.2015.07.037 26226576

[9] Heather Hampel JA, Stephens E, Pukkala R, Sankila LA, Aaltonen J-P, Mecklin (2005). Albert De La Chapelle. Cancer risk in hereditary nonpolyposis colorectal cancer syndrome: later age of onset. Gastroenterology.

[10] Suraweera N, Duval A, Reperant M (2002). Evaluation of tumor microsatellite instability using five quasimono- morphic mononucleotide repeats and pentaplex PCR[J]. Gastroenterology.

[11] Dedeurwaerdere F, Claes KB, Van Dorpe J, Rottiers I, Van der Meulen J, Breyne J, Swaerts K, Martens G (2021). Comparison of microsatellite instability detection by immunohistochemistry and molecular techniques in colorectal and endometrial cancer. Sci Rep.

[12] Leiman DA, Cardona DM, Kupfer SS, Rosenberg J, Bocsi GT, Hampel H, American Gastroenterological Association Quality Committee and the College of American Pathologists Quality Payment Measure Committee (2022). American Gastroenterological Association Institute and College of American Pathologists Quality Measure Development for Detection of Mismatch Repair Deficiency and Lynch Syndrome Management. Gastroenterology.

[13] Zhang ZH, Su PY, Hao JH (2013). The role of preexisting diabetes mellitus on incidence and mortality of endometrial cancer: a meta- analysis of prospective cohort studies[J]. Int J Gynecol Cancer.

[14] Deng L, Gui Z, Zhao L (2012). Diabetes mellitus and the incidence of colorectal cancer: an updated systematic review and metaanalysis[J]. Dig Dis Sci.

[15] Tianyi YE, Hongwen YAO, Lingying WU, Gongyi ZHANG (2015). Double primary carcinoma of endometrial carcinoma and colorectal carcinoma: retrospective analysis of 34cases and discussion of its relationship with Lynch syndrome[J]. Chin J Clin Oncol.

[16] Lancaster JM, Powell CB, Kauff ND (2007). Society of Gynecologic Oncologists Education Committee statement on risk assessment for inherited gynecologic cancer predispositions[J]. Gynecol Oncol.

[17] Win AK, Lindor NM, Winship I (2013). Risks of colorectal and other cancers after endometrial cancer for women with Lynch syndrome[J]. J Natl Cancer Inst.

[18] Win AK, Lindor NM, Young JP (2012). Risks of primary extracolonic cancers following colorectal cancer in Lynch syndrome[J]. J Natl Cancer Inst.

[19] Hong LIU, Dengfeng WANG, Yang LIU, Ying FAN, Wen DIE, Yang XIANG, Guonan ZHANG. Interpretation of the Chinese Expert Consensus on Screening and Prevention of Lynch Syndrome-Associated Endometrial Cancer (2023 Edition)[J]. Tumor Prevention and Treatment,2023,36(3):194–9.10.3969/j.issn.1674-0904.

[20] Buchanan D, Rosty C, Clendenning M, Spurdle A, Win AK (2014). Clinical problems of colorectal cancer and endometrial cancer cases with unknown cause of tumor mismatch repair deficiency (suspected Lynch syndrome). Appl Clin Genet.

[21] Morak M, Heidenreich B, Keller G, Hampel H, Laner A, de la Chapelle A (2014). Holinski-Feder, E. Biallelic MUTYH mutations can mimic Lynch Syndrome. Eur J Hum Genet.

[22] Jansen AM, van Wezel T, van den Akker BE, Ventayol Garcia M, Ruano D, Tops CM, Wagner A, Letteboer TG, Gómez-García EB, Devilee P, Wijnen JT, Hes FJ, Morreau H (2016). Combined mismatch repair and POLE/POLD1 defects explain unresolved suspected Lynch syndrome cancers. Eur J Hum Genet.

[23] Gueneau E, Dherin C, Legrand P, Tellier-Lebegue C, Gilquin B, Bonnesoeur P, Londino F, Quemener C, Le Du MH, Márquez JA, Moutiez M, Gondry M, Boiteux S, Charbonnier JB (2013). Structure of the MutLα C-terminal domain reveals how Mlh1 contributes to Pms1 endonuclease site. Nat Struct Mol Biol.

[24] Olkinuora A, Gylling A, Almusa H, Eldfors S, Lepistö A, Mecklin J-P, Nieminen TT, Peltomäki P (2020). Molecular basis of Mismatch Repair Protein Deficiency in Tumors from Lynch suspected cases with negative germline test results. Cancers.

[25] Loconte DC, Patruno M, Lastella P, Di Gregorio C, Grossi V, Forte G, Ingravallo G, Varvara D, Bagnulo R, Simone C, Resta N, Stella A (2014). A rare MSH2 mutation causes defective binding to hMSH6, normal hMSH2 staining, and loss of hMSH6 at advanced cancer stage. Hum Pathol.

[26] Kariola R, Raevaara T, Lönnqvist K. Functional analysis of MSH6 mutations linked to kindreds with putative hereditary non-polyposis colorectal cancer syndrome. Hum Mol Genet. 2002;11:1303–10. & Nyström-Lahti, Minna 10.1093/hmg/11.11.1303 12019211

[27] Peltomäki P, Nyström M, Mecklin JP, Seppälä TT (2023). Lynch Syndrome Genetics and Clinical implications. Gastroenterology.

[28] Mensenkamp AR, Vogelaar IP, van Zelst–Stams WAG, Goossens M, Ouchene H, Hendriks–Cornelissen SJB, Kwint MP, Hoogerbrugge N, Nagtegaal ID, Ligtenberg MJ (2014). L. somatic mutations in MLH1 and MSH2 are a frequent cause of Mismatch-Repair Deficiency in Lynch Syndrome-Like Tumors. Gastroenterology.

