# Supplementary material for: Rare germline mutation and MSH2-&MSH6 + expression in a double primary carcinoma of colorectal carcinoma and endometrial carcinoma: a case report
Source: Diagn Pathol. 2024 Jan 31;19:25. doi: 10.1186/s13000-024-01447-8 (PMC10829171; doi:10.1186/s13000-024-01447-8)
Supplement: Supplementary file 1 — Supplementary Material 1 [file 13000_2024_1447_MOESM1_ESM.docx]

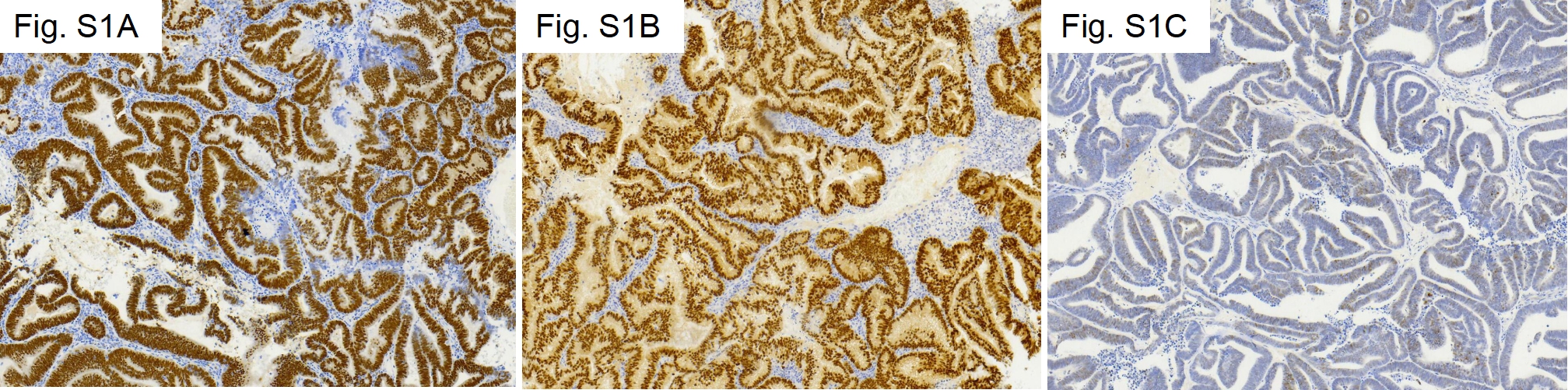


Supplementary Fig. 1, Moderate differentiation endometrioid adenocarcinoma in the uterus, immunohistochemical ER, PR, and P53 protein. ER (S1A) and PR (S1B) were strongly expressed in the uterus sample. P53 (S1C) was moderately expressed.


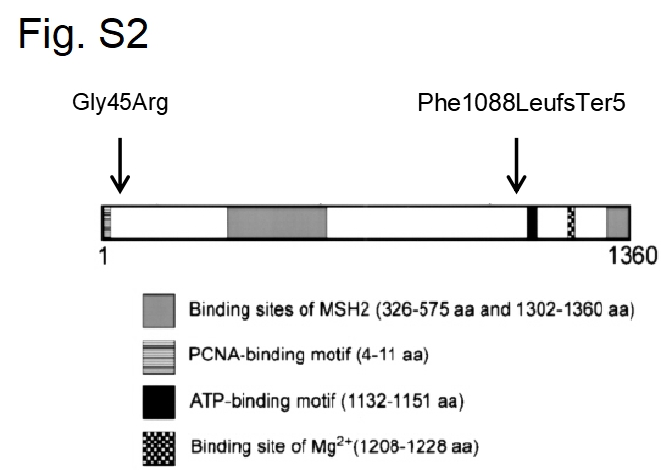


Supplementary Fig. 2, Schematic representation of the functional domain of the hMSH6 protein (adapted from Lützen et al [26]). Phe1088LeufsTer5 is located in exon 5 and results in a premature termination codon, predicted to cause a truncated or absent MSH6 protein due to nonsense mediated decay.
